# Supplementary material for: Mutational hotspots lead to robust but suboptimal adaptive outcomes in certain environments
Source: Microbiology (Reading). 2023 Oct 10;169(10):001395. doi: 10.1099/mic.0.001395 (PMC10634368; doi:10.1099/mic.0.001395)
Supplement: Supplementary material 1 [file mic-169-1395-s001.pdf]

SUPPLEMENTARY MATERIALS

**Table S.1. Strains used in this study**

| Strain | Specification | Growth Condition | Source |
|---|---|---|---|
| **Pf0-2x** | Immotile derivative of *Pseudomonas fluorescens* Pf0-1. Transposon insertion disruption of *fleQ*. | Ampicillin 100 µg/µl or Streptomycin 50/250 µg/µl | (1) |
| **Pf0-2x-sm** | Transposon insertion disruption of *fleQ*, and 6 synonymous base changes in *ntrB* generating a mutational hotspot. | Ampicillin 100 µg/µl or Streptomycin 50/250 µg/µl | (2) |
| **Pf0-1 delta fleQ** | Deletion of *fleQ*. Functionally equivalent to Pf0-2x. | - | Gifted by Mark Silby |
| **Pf0-2x-sm *ntrB* A289C *glmS*::Kan$^R$** | Evolved motile version of Pf0-2x-sm with a kanamycin resistance cassette inserted into the *glmS* locus via mini Tn7 transposition | Streptomycin 50/250 µg/µl + Kanamycin 50 µg/µl | This study |

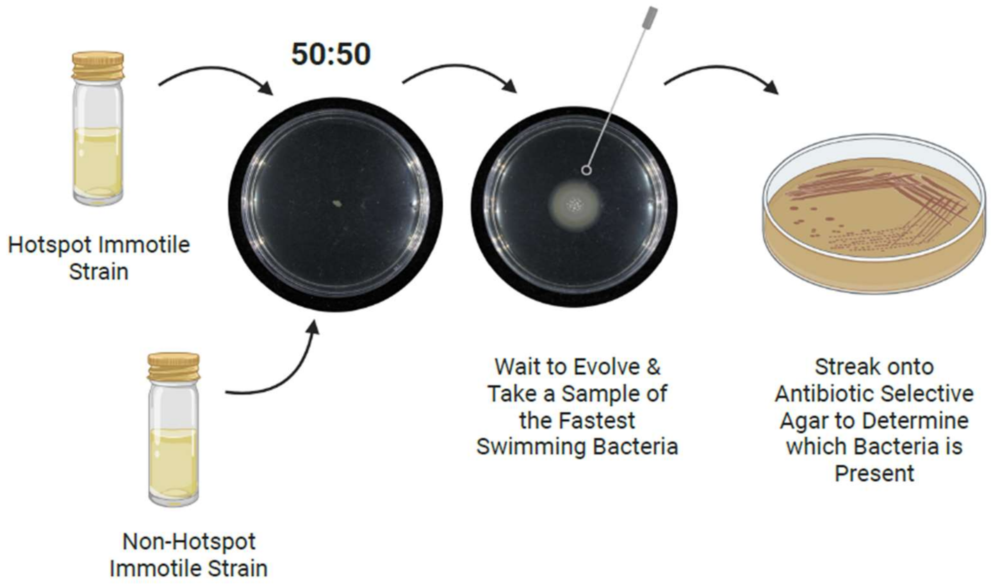


**Fig.S.1. Experimental workflow of evolutionary rescue competitions.** Created using BioRender.com

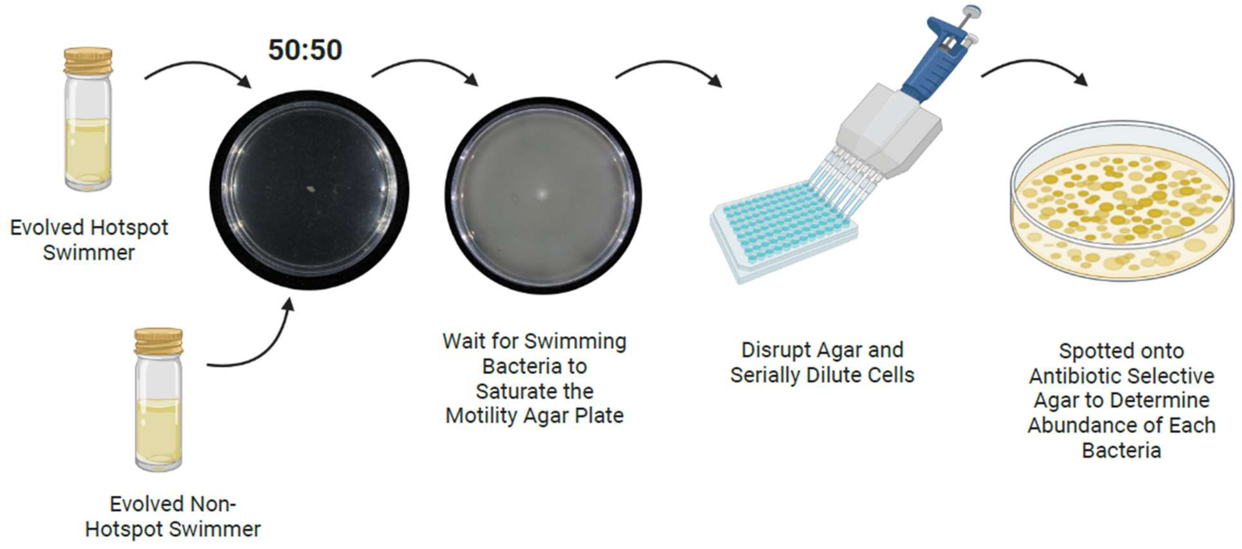


**Fig.S.2. Experimental workflow of swimming competitions.** Created using BioRender.com

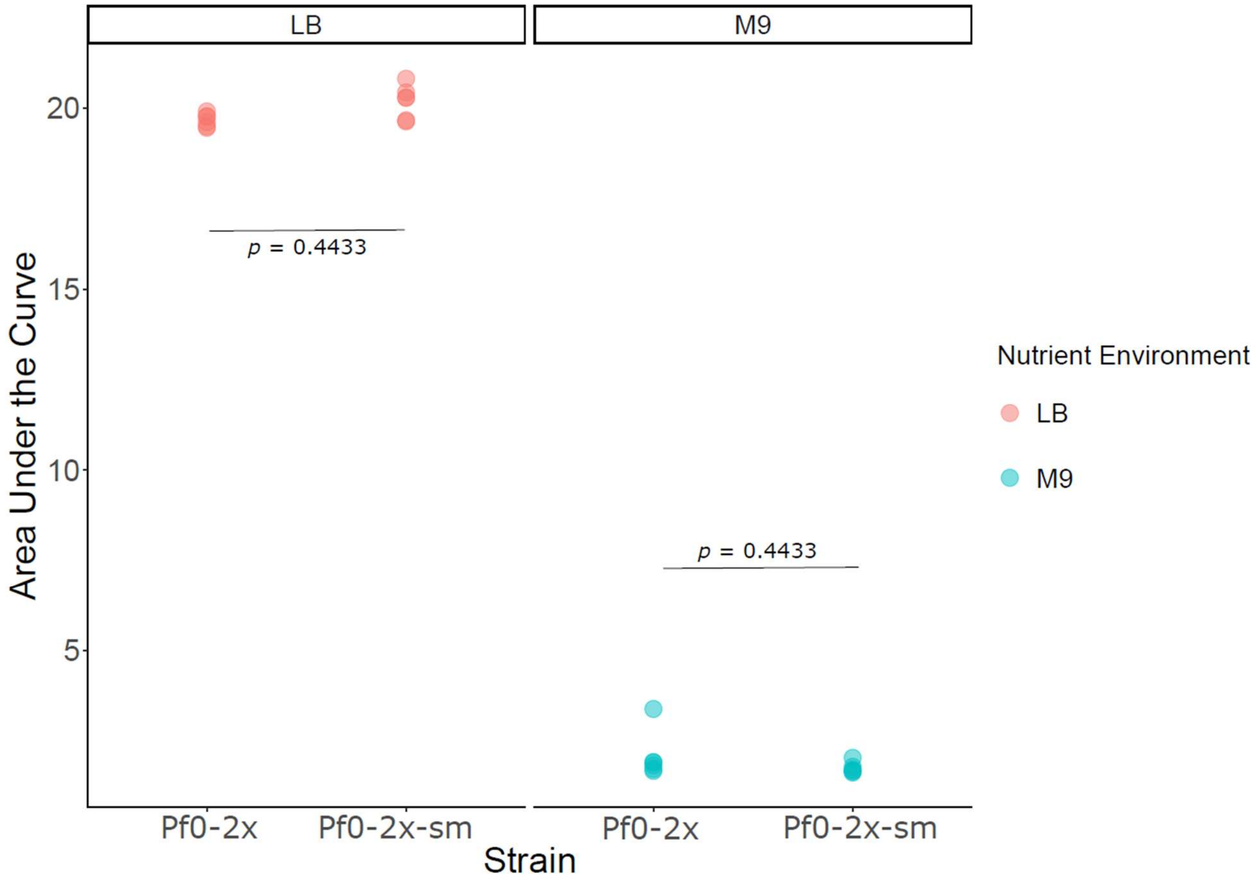


**Fig.S.3 Measure of metabolic growth of Pf0-2x and Pf0-2x-sm.**

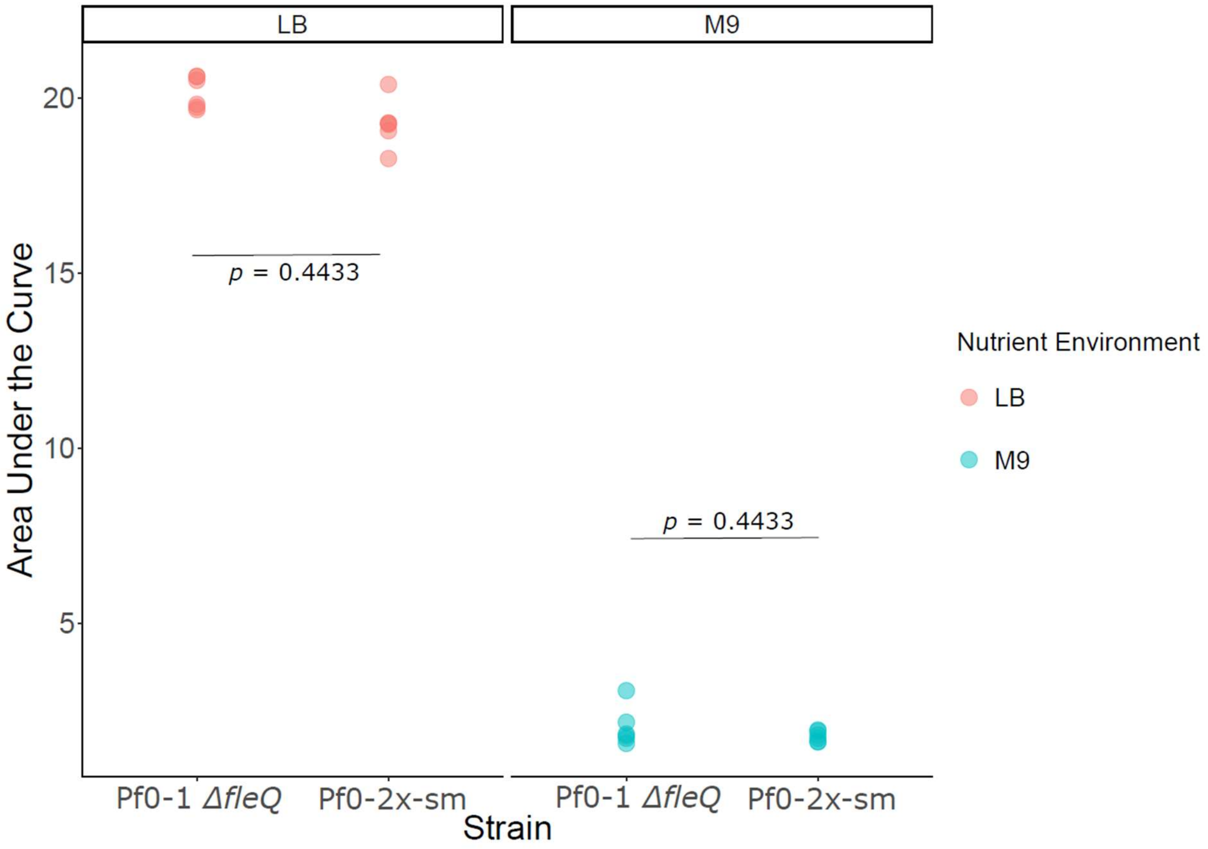


**Fig.S.4 Measure of metabolic growth of Pf0-2x-sm and Pf0-1 Δ*fleQ*.**

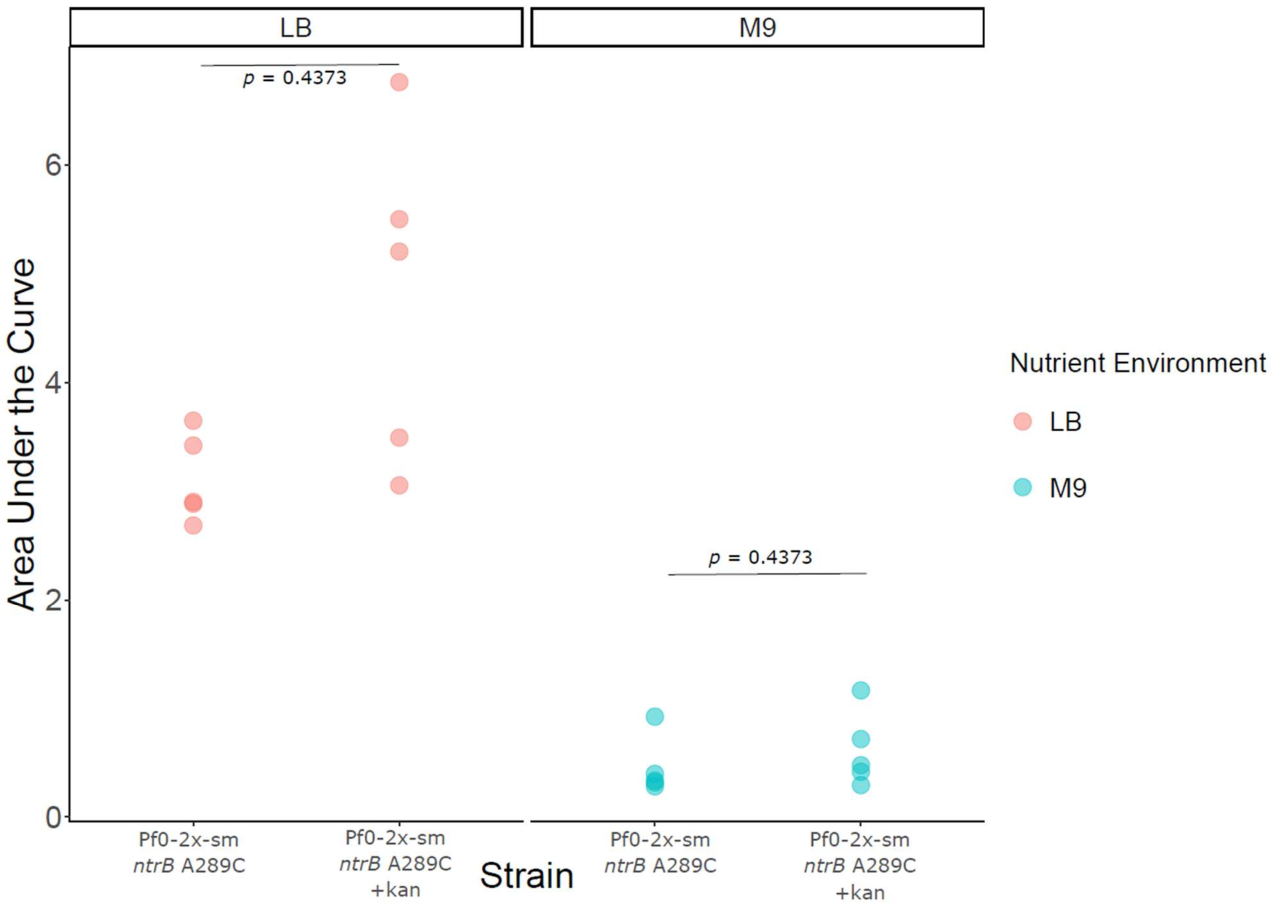


**Fig.S.5 Measure of metabolic growth of Pf0-2x-sm *ntrB* A289C and Pf0-2x-sm *ntrB* A289C *glmS*::Kan$^{R}$.**

(a)

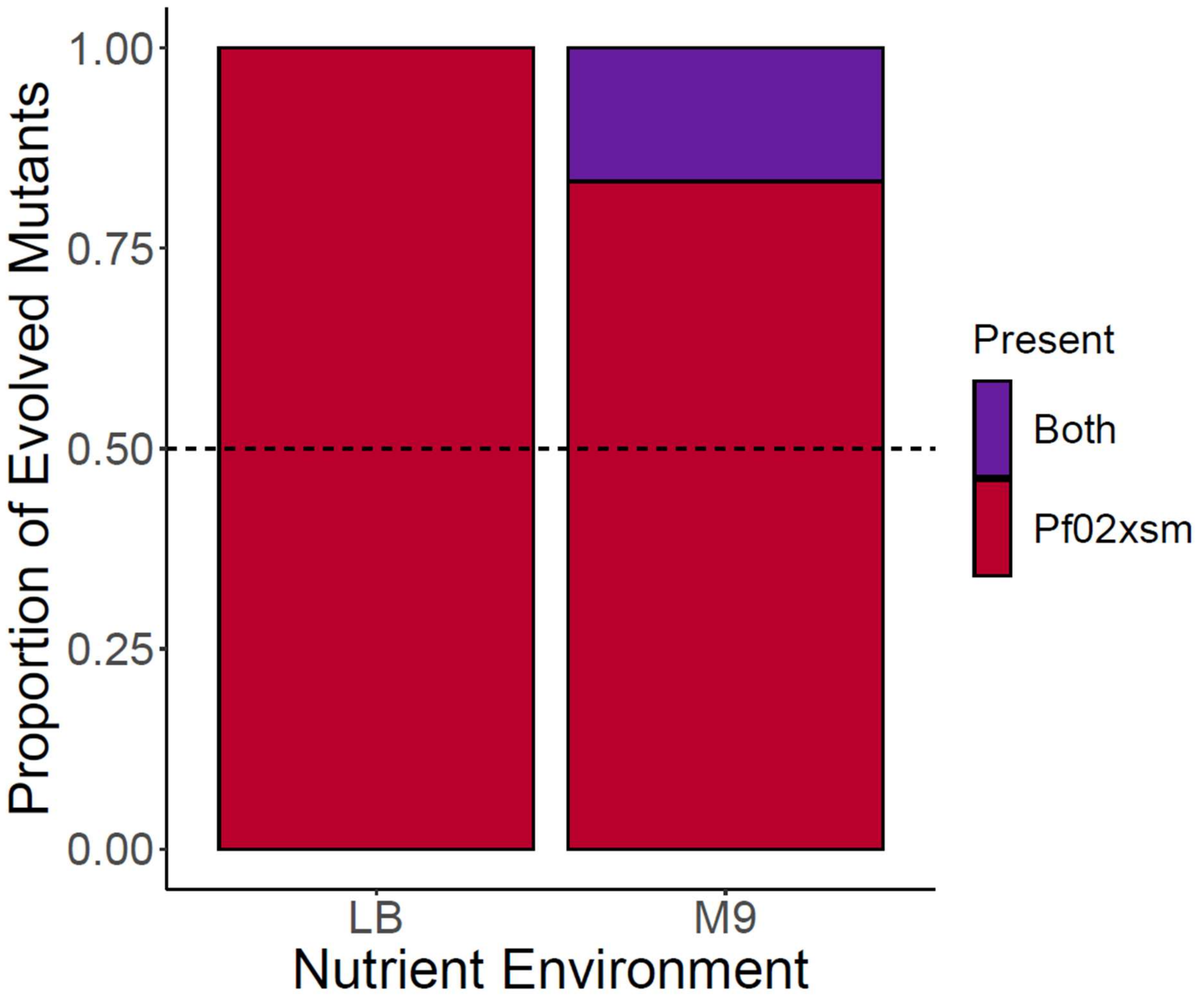


(b)

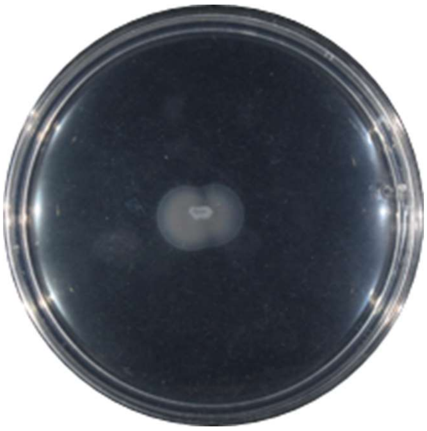

**Fig.S.6 Competitive evolution of a motility phenotype between the immotile hotspot and non-hotspot lines in complex and minimal media.**

The hotspot (Pf0-2x-sm) and non-hotspot (Pf0-1 *ΔfleQ*) lines were inoculated in equal cell numbers (50:50) in LB (*n*=6) and M9 (*n*=6) 0.25% motility agar plates and motility allowed to evolve. When a motility phenotype was restored, the edge of the motile zone was sampled

onto selective agar to determine which line had evolved first. **(a)** In the LB nutrient environment, the hotspot line was present at the leading edge in all replicates. In the minimal media environment M9, the hotspot line was present at the leading edge in 5 out of 6 replicates, but in one instance the non-hotspot line co-occurred at the leading edge of a second motile zone. The dashed black line crossing the y intercept at 0.5 indicates the expected values of each line if the competition outcome had been equal. **(b)** 0.25% M9 agar plate where the hotspot and non-hotspot lines evolved motility at the same time.

(a)

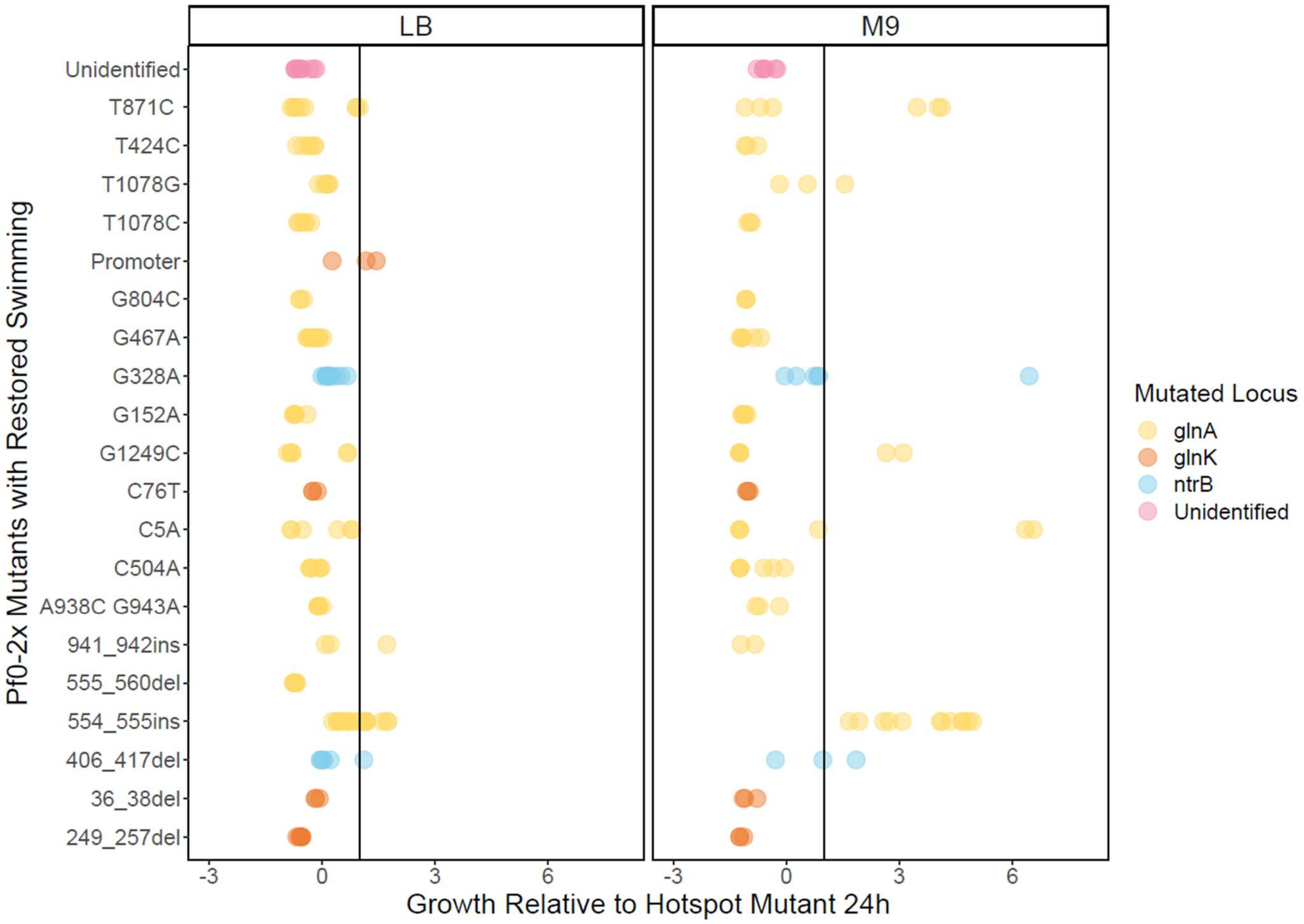

(b)

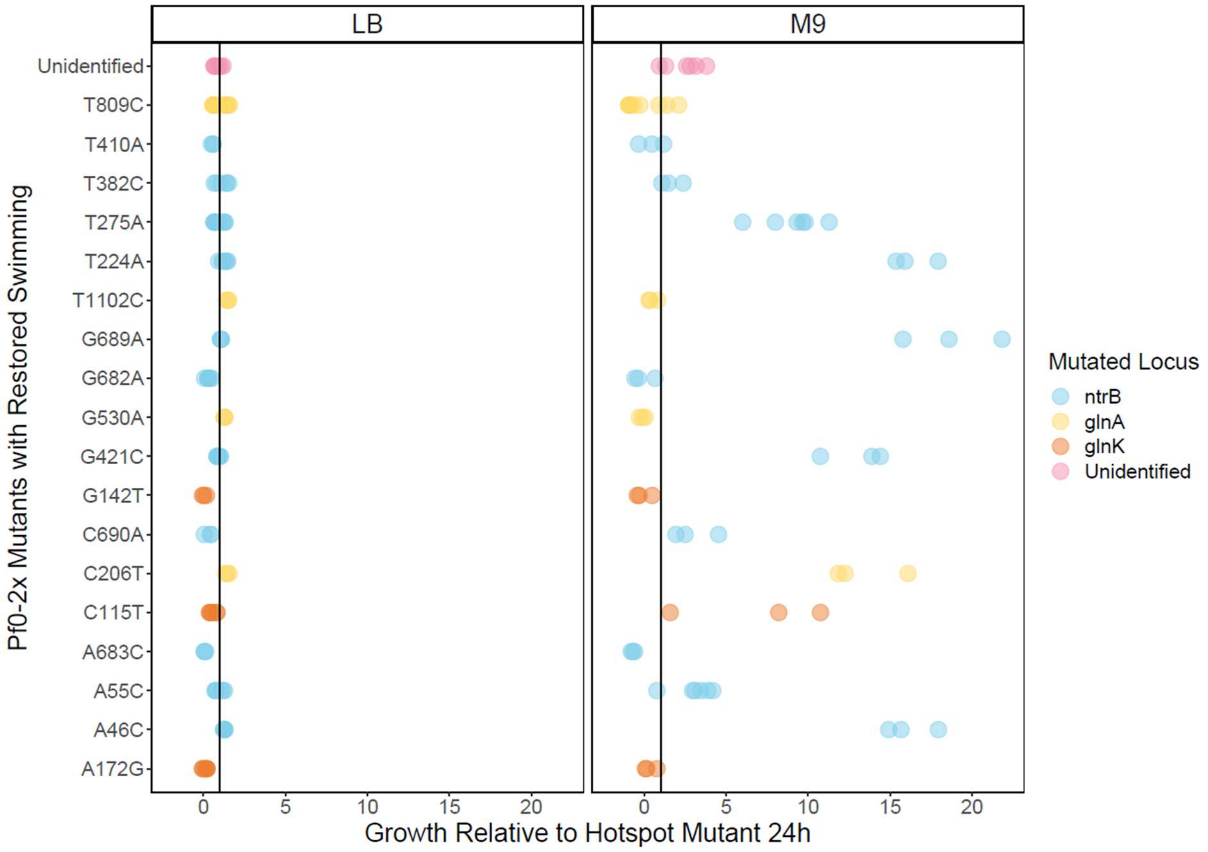


**Fig.S.7 LB and M9 growth assay of all Pf0-2x evolved mutants relative to the common hotspot mutant.** LB (a) and M9 (b) adapted Pf0-2x mutants (*n*=22, *n*=19) were assayed in LB and M9 in at least biological triplicate for 24h 180 rpm. The black line indicated the growth of the common hotspot mutant. Note that the x axis in the LB plot ranges from -3-8, while it ranges from -2-22 in the M9 plot.

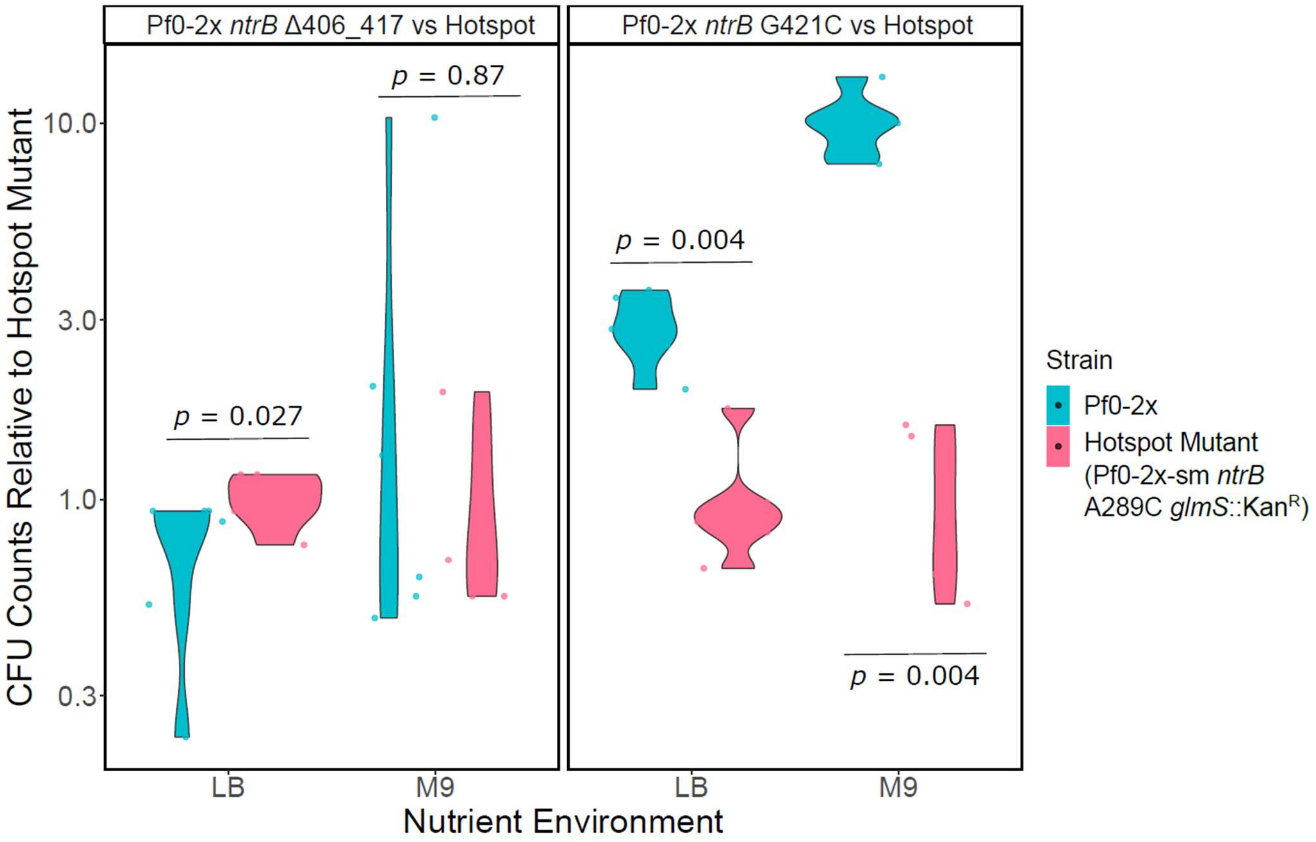


**Fig.S.8 The relative abundance of the non-hotspot fastest swimmers in competition with the common hotspot mutant in shaking broth.** The fastest non-hotspot (Pf0-2x) evolved lines (LB evolved Pf0-2x *ntrB* Δ406-417 and M9 evolved Pf0-2x *ntrB* G421C) were competed 50:50 against the hotspot mutant (Pf0-2x-sm *ntrB* A289C *glmS*::Kan$^R$) in both shaking LB and M9 broth for 24 h (*n*=6 for each competition). Colony forming units (CFUs) are given as relative to the hotspot evolved strain on a log scale.

**Table S.2. Primers used in this study**

| Primer Name | Primer Sequence | Function |
|---|---|---|
| **ntrB_Pf01_F** | GACGATCCACACGGTTTC | Amplifies *ntrB* gene in Pf0-1/Pf0-2x/Pf0-2x-sm |
| **ntrB_Pf01_R** | CCATGCTCCAAAAGAGGTC | Amplifies *ntrB* gene in Pf0-1/Pf0-2x/Pf0-2x-sm |
| **ntrB_1119_F** | GAGGTCCCAATGACCATCAG | Amplifies *ntrB* gene in SBW25/AR2 and Pf0-1/Pf0-2x/Pf0-2x-sm |
| **ntrB_1119_R** | GACGATCCAGACGGTTTCAC | Amplifies *ntrB* gene in SBW25/AR2 and Pf0-1/Pf0-2x/Pf0-2x-sm |
| **Pf01_GlnK_FW** | GTCATATTGTTTTCTCCTGGG | Amplifies *glnK* gene in Pf0-1/Pf0-2x/Pf0-2x-sm |
| **Pf01_GlnK_RV** | GTTTTCCTGGGGCAAGTC | Amplifies *glnK* gene in Pf0-1/Pf0-2x/Pf0-2x-sm |
| **Pf01_glnA_FW** | CTAATTCACCCGGAGGACAC | Amplifies *glnA* gene in Pf0-1/Pf0-2x/Pf0-2x-sm |
| **Pf01_glnA_RV** | AGGAGGCCTTTCTTTGTCAC | Amplifies *glnA* gene in Pf0-1/Pf0-2x/Pf0-2x-sm |

SUPPLEMENTARY REFERENCES

1. Robleto EA, López-Hernández I, Silby MW, Levy SB. Genetic analysis of the AdnA regulon in Pseudomonas fluorescens: Nonessential role of flagella in adhesion to sand and biofilm formation. J Bacteriol. 2003;185(2):453–60.

2. Horton JS, Flanagan LM, Jackson RW, Priest NK, Taylor TB. A mutational hotspot that determines highly repeatable evolution can be built and broken by silent genetic changes. Nat Commun 2021 121. 2021 Oct 19;12(1):1–10.
